# Molecular detection and antibiogram of Shiga toxin-producing *Escherichia coli* (STEC) from raw milk in and around Bahir Dar town dairy farms, Ethiopia

**DOI:** 10.1016/j.heliyon.2024.e28839

**Published:** 2024-04-03

**Authors:** Fanuel Bizuayehu Yihunie, Mequanint Addisu Belete, Gizachew Fentahun, Teshager Dubie

**Affiliations:** a College of Veterinary Medicine and Animal Science, Samara University, Semera, Ethiopia; b Department of Veterinary Laboratory Technology, College of Agriculture and Natural Resource, Debre Markos University, Debre Markos, Ethiopia; c Institute of Biotechnology, Addis Ababa University, Addis Ababa, Ethiopia

**Keywords:** Antimicrobial resistance, Bahir Dar, *Escherichia coli*, Milk, Shiga toxin, Virulence gene

## Abstract

Illnesses associated with consuming infected milk and milk products are a widespread problem in low and middle-income countries. Shiga toxin-producing *Escherichia* coli (STEC) is a bacterium commonly found in raw milk and causes foodborne diseases ranging from mild diarrhea to severe hemorrhagic colitis and hemolytic uremic syndrome. This study aimed to investigate the virulence gene and antimicrobial resistance profiles of Shiga toxin-producing *E. coli* strains isolated from raw milk in dairy farms in and around Bahir Dar town. Raw milk samples (n = 128) collected from December 2021 to July 2022 were cultured, and *E. coli* strains were isolated using standard methods. Shiga toxin-producing *E. coli* strains were identified genotypically by the presence of the virulence markers using a single-plex polymerase chain reaction. The antibiotic susceptibility testing of Shiga toxin-producing *E. coli* isolates was done by the agar disk diffusion method. In total, 32 *E. coli* isolates were recovered from milk samples from lactating animals. PCR screening of these isolates resulted in 19 (59.3%) positives for Shiga toxin-producing *E. coli*. The *stx2* gene was detected in 53% of cases, followed by *stx1* (31%) and *eae* (16%. The STEC isolates were highly sensitive to ciprofloxacin (94.7%) and kanamycin (89.5%), while exhibiting significant resistance to amoxicillin (89.5%) and streptomycin (73.7%). The present study points out the occurrence of virulent and antibiotic-resistant Shiga toxin-producing *E*. *coli* strains in raw milk that could pose a potential risk to public health. Further analysis by whole genome sequencing is necessary for an in-depth assessment and understanding of their virulence and resistance factors. Moreover, large-scale studies are needed to identify the prevalence and potential risk factors and to prevent the spread of antibiotic-resistant STEC strains in the milk production chain.

## 1 Introduction

Outbreaks of human diseases can arise from consuming raw animal products (meat and dairy products), vegetables, spoiled fruit products, and water [1,2]. Milk is a significant source of nutrients for human health and development, but it is also a potential source of microbial agents that pose severe illness to milk consumers. The quality, quantity, and safety of milk in low-income settings are minimal due to a lack of knowledge of modern production and unsafe milk handling. In addition to the milk handlers' hygienic status, the environment contributes to milk-borne disease occurrence [3].

In Ethiopia, dairy production is marginal and more of the traditional type [4]. Milk is produced from cattle, goats, camels, and sheep. Recently, there has been a noticeable increase in the utilization of sheep milk for human consumption within the pastoral areas and the southern regions of Ethiopia, drawing attention to its economic potential [5,6]. The foremost milk production sources were cattle, which accounted for the dominant percentage (81.2%) of the total national annual milk output, followed by goats (7.9%), camels (6.3%), and sheep (4.6%) [7].

Previous reports in Ethiopia showed a high level of *E. coli* contamination in foods of animal origin, with an overall pooled prevalence of 15% [8]. *E. coli* strains are recognized as the cause of colibacillosis in food animals and severe human diseases like hemorrhagic colitis and hemolytic uremic syndrome [9,10]. Shiga toxin-producing *E. coli* (STEC) are the most common zoonotic food and waterborne pathogens responsible for large outbreaks and numerous sporadic infections. The major virulence factors implicated in STEC infection are potent Shiga toxins: Shiga toxins 1 and 2 (Stx1 and Stx2), which inhibit protein synthesis as well as act in cell signal transduction and immune modulation, causing pro-inflammatory and pro-apoptotic responses [[11], [12], [13]].

Food-borne pathogens are still the leading cause of disease and death in low and middle-income countries (LMIC). It has significant losses in the economy of LMIC in addition to the lives of humans, costing billions of dollars in medical care [14]. Keeping the hygiene of the premises and the personnel plays an indispensable role in the prevention of microbial contamination in the milk [15,16].

Antibiotic therapy is a choice to alleviate the illness due to food-borne infections like *E. coli*. However, the misuse of antibiotics and the drug residue in the bulk of milk has contributed to the development of antibiotic resistance [17]. One of the most critical issues in the globe is the evolution, rising occurrence, and wide spreading of pathogenic bacteria that are resistant to a wide range of antibiotics [18]. Multidrug-resistant (MDR) strains of *E. coli* have emerged by acquiring resistance genes via different gene transfer methods or genetic changes due to mutation [19].

Milk producers in rural areas of Ethiopia often lack knowledge about microbial contamination during milk production, collection, and handling [20]. Detecting microbes in samples taken during these phases is an indication of contamination originating from the animal or the environment [21,22]. In the study area, raw milk consumption is a common practice since it serves as an immediate food supply that could alleviate hunger. Milk is consumed immediately after milking or stored for some time without processing. Though milk is the most complete food, it must be pasteurized before consumption to avoid milk-borne infection and intoxication [23]. Community awareness regarding the harmful effects of consuming raw milk is often neglected. No previous studies have been reported about the level of STEC contamination in raw cow's milk in dairy farms of the study area. Therefore, the study aims to determine the occurrence and antibiogram of STEC pathotype in raw milk in and around Bahir Dar town. Hence, this study demonstrated the existence of STEC contaminants and their antibiotic resistance in raw milk, which could pose health hazards to consumers.

## 2 Materials and methods

### 2.1 Study area

**Fig. 1:**
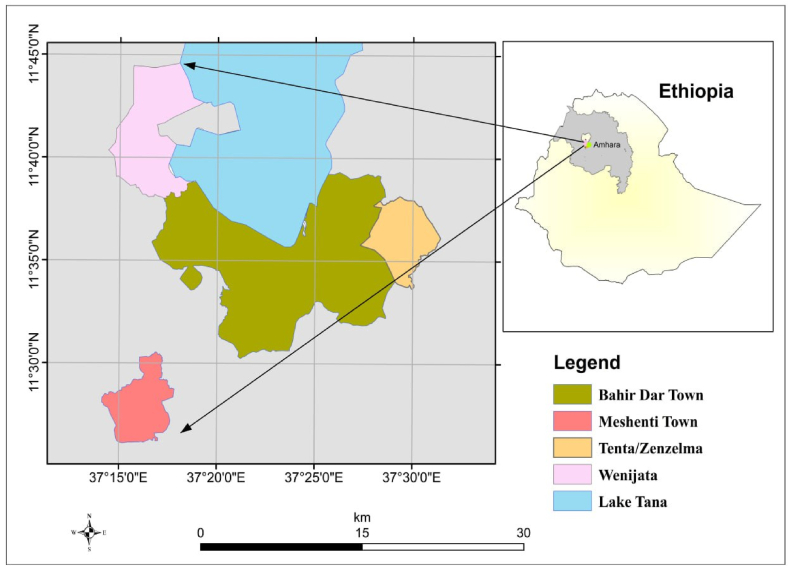
Map of the study area in and around Bahir Dar town, Amhara regional state, Ethiopia.

This study was conducted in and around Bahir Dar town, the seat of Amhara regional state, Northwest Ethiopia, from December 2021 to July 2022 (Fig. 1). The city is 565 km from Addis Ababa, the capital of Ethiopia. The town is densely populated, with an urban and peri-urban lifestyle. Dairy production is practiced in and around the town, supporting the livelihood of the inhabitants of Bahir Dar. The mean annual rainfall of the city is about 596 mm, with a bimodal rainy season distribution of 70%, which occurs from June to September and 30% from March to April [24].

### 2.2 Study population

The animals enrolled in the study were apparently healthy, comprising both local and cross-breed lactating cows reared in middle-scale and small-holder dairy farms. The cows were hand-milked twice, with a 12-h interval between each milking. The study did not consider information such as the stage of lactation, parity, level of milk production, body conditions, farm management, and previous history of mastitis.

### 2.3 Study design and sampling methods

A cross-sectional study design was employed to investigate the occurrence of STEC pathotypes and determine their antibiotic susceptibility patterns. Purposive sampling was used to select dairy farms and collect milk samples from lactating cows. The presence of lactating cows was the basis of farm and cow selection. Overall, 128 raw milk samples from 128 animals (32 from Bahir Dar town, 38 from Meshenti town, 30 from Tenta/Zenzelma, and 28 from Wenijata) were collected from 30 dairy farms, including 21 smallholders (Meshenti town/n = 10), Wenijata/n = 6), and Tenta/Zenzelma/n = 5) and nine medium scales (Bahir Dar town (n = 9). All quarter milk from each animal was collected and pooled into a single bottle (a single sample contains the four-quarter milk of a single cow). The samples were collected aseptically by disinfecting the cow's udder and washing the milking hand with soap and 70% alcohol to avoid external contamination. A sterile sampling bottle was used for milk collection. Finally, the samples were processed at the microbiology laboratory department of the Amhara Public Health Institute (APHI).

### 2.4 Bacteriological isolation and identification

The isolation of *E. coli* was conducted based on the protocol described by Quinn [25]. The milk samples were cultured at 37 °C for 24 h using nutrient agar (HiMedia, India). After 24-h periods of incubation, Gram's reaction was performed by taking pure suspected colonies and examining for Gram-negative characteristics of the bacteria. Colonies suspected of Gram-negative were further inoculated into MacConkey agar (HiMedia, India) plates to confirm that the bacterium is Gram-negative and capable of fermenting lactose [26]. Then pinkish colonies on the MaConkey agar (lactose fermenter) were subcultured into eosin methylene blue agar (EMB) (HiMedia, India) to appreciate the characteristic metallic sheen appearance of *E. coli*. Afterward, the presumptive *E. coli* isolates were further subjected to biochemical tests, including the catalase test, oxidase test, Indole production, Methyl Red - Voges-Proskauer (MR-VP) test, citrate utilization test, triple sugar iron agar (TSI) test, as per the procedures outlined [22,27].

### 2.5 Molecular detection of virulence genes in *E. coli* isolates

The PCR assays were performed using four specific primers (Table 1), targeting the *stx1*, *stx2*, *eae*, and *ribo157* genes as previously described [24,28]. The DNA was extracted using a Qiagen DNeasy Blood and Tissue Kit based on the manufacturer's instructions (Qiagen, Germantown, MD, USA). The purity and concentration of the extracted DNA were measured using a Nanodrop 2000/2000C Spectrophotometer (Thermo Scientific™, USA). A ratio of 1.6–2.0 margin is accepted as a pure DNA [29]. Then, the PCR test was employed in a final 25 μl reaction volume, of which 2.5 μl of PCR buffer with 7.5 mM MgCl2 (10X, HiMedia), 1 μl of dNTP (100 Mm, HiMedia), 0.5 μl of forward and reverses primers each (Bioneer), 0.5 μl of Taq DNA polymerase (5 U/μl, Solis BioDyne), 3 μl of template DNA and 17 μl of nuclease-free water. Reference strains containing the virulence genes and nuclease-free water were used as positive and negative controls, respectively. The amplification process was carried out in Prima 96 plus Thermal Cycler (Himedia Laboratories, India), and the PCR condition was set with an initial denaturation at 95 °C followed by 35 cycles of denaturation, annealing, and elongation with specified Temperature and time for each specific gene. Then the reaction was held at 72 °C for 10 min for a final extension before cooling at 4 °C (Table 1).

**Table 1:** PCR primer sequences, conditions, and products used for the detection of virulence genes.

Target gene	Primer sequence	PCR conditions						No of cycles	PCR product size (bp)	References
		Denaturation		Annealing		Extension				
		T^0^	Time	T^0^	Time	T^0^	Time			
*stx1*	F:ATCAGTCGTCACTCACTGGT R:CTGCTGTCACAGTGACAAA	95^0^c	40sec	55^0^c	30sec	72^0^c	1min	35	110	[24]
*stx2*	F:CAACACTGGATGATCTCAGC R:CCCCCTCAACTGCTAATA	95^0^c	40sec	55^0^c	30sec	72^0^c	1min	35	350	[24]
*rfbo157*	F:AAGATTGCGCTGAAGCCTTTG R:CATTGGCATCGTGTGGACAG	94^0^c	30sec	66^0^c	30sec	72^0^c	30sec	35	479	[30]
*ea*e	F:AAACAGGTGAAACTGTTGCC R:CTCTGCAGATTAACCTCTGC	95^0^c	40sec	55^0^c	30sec	72^0^c	1min	35	490	[28]

T^0^ = Temperature, sec = second, min = minute.

Gel electrophoresis was done for PCR products using 1.5% (w/v) agarose gel (Bio Basic Inc. Canada) stained with 10 mg/ml gel red. Electrophoresis was conducted at 120 V for 60 min via an electrophoresis apparatus (EC 2060, USA) containing 1X TAE buffer (40 mM Tris, 1 mM EDTA, and 20 mM glacial acetic acid, pH 8.0). The band sizes of the PCR products were visualized under a UV *trans*-illuminator and gel documentation system (BioRAD, USA). A 100-bp molecular weight DNA ladder (Solis BioDyne, Estonia) was loaded into the first lane of the gel to determine the size of the amplified PCR product.

### 2.6 Antibiotic susceptibility tests

All PCR-confirmed STEC isolates were tested for antibiotic sensitivity. The susceptibility test was employed according to the Kirby-Bauer disc diffusion method using eight (amoxicillin (20 μg), ciprofloxacin (5 μg), streptomycin (10 μg), gentamicin (10 μg), ampicillin (25 μg), chloramphenicol (30 μg) (HiMedia, India), kanamycin (10 μg), and tetracycline (10 μg) (Oxoid, England)), commercially available antibiotic-impregnated disks. The antibiotics were selected based on the treatment of choice for *E. coli* strains. Antibiotics were also selected based on their availability and frequent utilization for treating disease in the area. Mueller Hinton (MHA) agar media (Oxoid, England) and a 0.5 Mac-Farland standard suspension of the bacteria were prepared to culture and adjust the density of the bacterial suspension, respectively. The result of the zone of inhibition was measured and interpreted as sensitive, intermediate, or resistant according to the Clinical and Laboratory Standards Institute (CLSI) guidelines [24].

### 2.7 Data management and analysis

The raw data was recorded and coded using Microsoft Excel (Microsoft ® Office Excel, 2016). The occurrence of virulence genes and the determination of antimicrobial patterns were analyzed using descriptive statistics. The Statistical Package for Social Sciences (SPSS), version 20 (IBM, USA), was used for analyzing the data.

## 3 Results

**Fig. 2:**
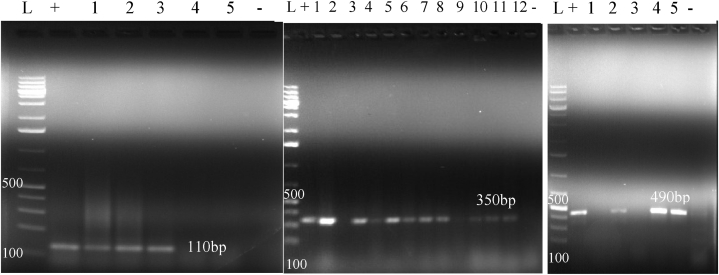
Gel electrophoresis result of PCR products for *stx1* (110bp), *stx2* (350bp), and *eae* (490bp) virulent genes. L is for DNA ladder (100bp), + (positive control), - (negative control), and the numbers representing the samples (supplementary file 1).

From the total 128 sampled milk, 32 (25%) were positive for *E. coli* by conventional bacterial culturing and biochemical profiling. All *E. coli* isolates were molecularly tested for virulence genes in addition to their phenotypic identification. The result revealed that 19 (59.3%) of the isolates harbored one or more virulent genes (Fig. 2). Of which, 6 (31%) were positive for *stx1*, 10 (53%) were positive for *stx2*, and 3 (16%) were positive for *eae* genes. However, none of the isolates contained the *rfbo157* gene (Table 2).

**Table 2:** Distribution of phenotypic and PCR-based results related to a study area and farm type among 128 raw milk samples, in and around Bahir Dar town, Ethiopia.

Parameters	Categories		Phenotypic results	PCR detected virulent genes			
		No. of each samples	No. of positive (%)	*Stx1* (%)	*Stx2* (%)	*eae* (%)	*rfbo*157 (%)
**Study area**	Bahir Dar town	32	4 (12.5)	1 (16.7)	2 (20)	0 (0)	0 (0)
	Meshenti town	38	10 (31.2)	2 (33.3)	3 (30)	1 (33.3)	0 (0)
	Tenta/Zenzelma	30	11 (34.4)	2 (33.3)	4 (40)	2 (66.7)	0 (0)
	Wenijata	28	7 (21.9)	1 (16.7)	1 (10)	0 (0)	0 (0)
	Total	128	32 (100)	6 (100)	10 (100)	3 (100)	0 (0)
**Farm type**	Smallholder	96	28 (87.5)	5 (83.3)	8 (80)	3 (100)	0 (0)
	Medium scale	32	4 (12.5)	1 (16.7)	2 (20)	0 (0)	0 (0)
	Total	128	32 (100)	6 (100)	10 (100)	3 (100)	0 (0)

The antibiogram results revealed that the most common antibiotic resistance was observed against amoxicillin (89.5%), gentamycin (57.9%), tetracycline (57.9%), and ampicillin (52.6%). On the other hand, the highest susceptibility was recorded for ciprofloxacin (94.7%), kanamycin (89.5%), and chloramphenicol (84.2%) (Table 3).

**Table 3:** Antibiotic susceptibility profiles of STEC isolates.

Antimicrobial Agents	Zone diameter breaking point (mm)			S N (%)	I N (%)	R N (%)
	S	I	R			
Amoxicillin (20 μg)	≥18	14–17	≤13	0 (0)	2 (10.5)	17 (89.5)
Ciprofloxacin (5 μg)	≥26	22–25	≤21	18 (94.7)	1 (5.3)	0 (0)
Ampicillin(10 μg)	≥17	14–16	≤13	7 (36.9)	2 (10.5)	10 (52.6)
Streptomycin (10 μg)	≥15	12–14	≤11	1 (5.3)	4 (21)	14 (73.7)
Kanamycin (30 μg)	≥18	14–17	≤13	17 (89.5)	2 (10.5)	0 (0)
Tetracycline (30 μg)	≥15	12–14	≤11	3 (15.8)	5 (26.3)	11 (57.9)
Chloramphenicol (30 μg)	≥18	13–17	≤12	16 (84.2)	3 (15.8)	0 (0)
Gentamycin (10 μg)	≥15	13–14	≤12	5 (26.3)	3 (15.8)	11 (57.9)

S = susceptible, I = intermediate, R = resistant, N = number.

Interpretation of the result is based on CLSI standards [31].

## 4 Discussion

In this study, the overall isolation rate of *E. coli* was 32 (25%) through conventional bacteriological analysis. Among 32 presumptive isolates, 19 (59.3%) were PCR confirmed to the virulent genes of STEC. The variation between bacteriological and molecular results is due to the genetic variability of *E. coli* strains, which exhibit similar phenotypic characteristics [32]. In addition, the difference may arise from the techniques employed in identifying the characteristic features of the bacteria [28]. Furthermore, the difference in detection rates between culture-based and molecular methods in the present study may be because the molecular test only targets specific strains, specifically STEC [33].

The detection of virulent genes in the study is close to the finding of Mohammadi et al. [34] (56%) in Iran but showed a higher detection rate than the study of Khan et al. [28] (10%) in India, Gilmour et al. [35] (5%) in Canada, Cedillo et al. [36] (7%) in Norway, Neher et al. [37] (26%) in India and Leclerc et al. [38] (16%) in France. The current study detected a relatively low rate of STEC, contradicted by the studies of Fadaei [39] in Iran and Lubote et al. [40] in Tanzania, who reported 69% and 90.67%, respectively. However, the detection in our study is higher than reports by Mohanty et al. [26], who found 21% from India, and Lye et al. [2], who reported 8.75% from Malaysia. The variation in the recovery rate of the virulent gene may be due to the type/size of the sample, the season, agroecological zones, and the techniques employed for sample processing during the study. In the study, the *stx2* gene is more detected than *stx1* and *eae* genes, agreed with the studies conducted by Stephan et al. [41] and Rey et al. [42]. Studies in Germany indicated that the presence of neutralizing colostrum antibodies in bovine milk react more with Stx1 than Stx2-containing strains. This may account for the inhibition of Stx1 strains than of Stx2 strains [43]. The higher occurrence of Stx2 can be attributed to several factors, encompassing the biological characteristics of the toxins, variation in virulence, and genetic diversity of STEC strains. In this context, numerous studies have consistently suggested that *stx2* gene is generally considered more virulent, potent and linked to more severe clinical outcomes than *stx1* [[44], [45], [46]]. Additionally, STEC strains harboring the *stx2* genes may possess a selective advantage concerning colonization or survival within the host [47]. Moreover, Stx2-carrying STEC strains could exhibit a survival advantage in certain environmental conditions such as contaminated foods [48,49], all contributing to their higher detection rate compared to the *stx1* gene.

In the present study, *E. coli* isolates were found sensitive to ciprofloxacin (94.7%), kanamycin (89.5%), and chloramphenicol (84.2%). This sensitivity result agrees with the findings of Belete et al. [24] and Dejene et al. [15]. However, isolates were found resistant to amoxicillin (89.5%), in line with 64.9% resistance in a study conducted by Belete et al. [24] in Bahir Dar town. The resistance of *E. coli* to streptomycin (73.7%) was a comparable report with Dejene et al. [15] and Thaker et al. [11], presenting 70.37% and 57.89% resistance in central Ethiopia and India, respectively. Contamination of milk with antibiotic-resistance bacteria is multifaceted issue arising from diverse sources, including environmental factors, the action of milk handles and the materials employed in the milking process [27]. The underuse, misuse and excessive use of antibiotics is believed to contribute to the widespread prevalence of antibiotic-resistant bacteria [50]. Besides, antibiotic resistance could occur through various mechanisms such as gene alterations with in bacterial populations or through the horizontal transfer of resistance genes [51].

Tetracycline (57.9%) and gentamycin (57.9%) resistance in our study concur with the finding of [15], who found 59.26% resistance to tetracycline and 59.2% resistance to gentamycin in central Ethiopia. Ampicillin (52.6%) resistance in the current study is at a low-level resistance compared to the 100% resistance reported in different studies [11,15,17]. The variation in resistance and susceptibility may be due to selective pressure, the area the microbes investigated, the varieties of *E. coli* isolates, and the habit of antibiotic users. The overall resistance observed in *E. coli* isolates is due to mutation, acquiring resistant genes and selective pressure, different enzymes that can abort the activity of the antibiotics, and phenotypic changes [51]. Generally, this study confirmed the status of STEC in raw milk, which possibly has a harmful health impact on consumers. No significant studies are exploring the impact of pasteurization in reducing toxins from STEC isolates. Research conducted on Stx2 stability and pasteurization reported that Stx2 is heat stable at conventional pasteurization. However, pasteurization at 100^0^c for 5 min inactivated the toxin [52]. Antibiotic resistance is also an issue in the study area. However, the study has limitations on advanced molecular profiling of the target strain and resistant gene of the bacteria due to scarce resources. The study also has limitations in sampling pasteurized milk and identifying risk factors for STEC detection.

## 5 Conclusion

The study detected STEC strains in raw milk on farms in and around Bahir Dar town. Antibiotic resistance was recorded on the confirmed STEC isolates. The study highlights the risks of consuming raw milk. Therefore, due attention is needed while prescribing and using antibiotics. However, this study has limitations on small sample size and the molecular characterization of the target strain. Further comprehensive and molecular typing studies are recommended to deepen our understanding of the potential role of the strain in foodborne illness.

## Funding disclosure

There is no specific funding for this work.

## Data availability

Data will be made available on request.

## CRediT authorship contribution statement

**Fanuel Bizuayehu Yihunie:** Writing – review & editing, Writing – original draft, Validation, Methodology, Formal analysis, Data curation, Conceptualization, Investigation. **Mequanenet Addisu Belete:** Writing – review & editing, Writing – original draft, Software, Methodology, Formal analysis, Data curation. **Gizachew Fentahun:** Writing – original draft, Methodology, Formal analysis, Data curation, Software. **Teshager Dubie:** Validation, Methodology, Investigation, Formal analysis, Data curation.

## Declaration of competing interest

The authors declare that they have no known competing financial interests or personal relationships that could have appeared to influence the work reported in this paper.
