# Supplementary figures and images for: Molecular detection and antibiogram of Shiga toxin-producing Escherichia coli (STEC) from raw milk in and around Bahir Dar town dairy farms, Ethiopia
Source: Heliyon. 2024 Apr 3;10(7):e28839. doi: 10.1016/j.heliyon.2024.e28839 (PMC11004750; doi:10.1016/j.heliyon.2024.e28839)

**Supplementary file 1**

**Full, non-adjusted images of gels**

Stx1 gene


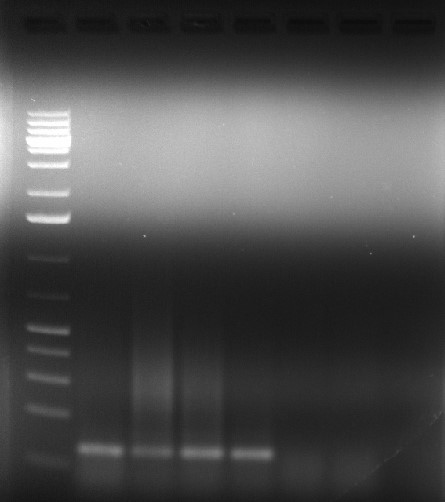


Stx2 gene


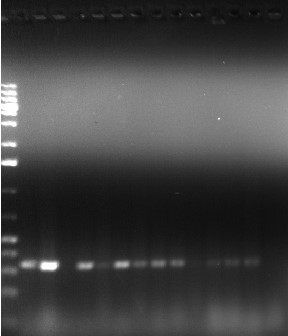

Supplement: Multimedia component 1 [file mmc1.docx]
